# How much time has passed? Ask your heart

**DOI:** 10.3389/fnbot.2014.00015

**Published:** 2014-04-09

**Authors:** Olga Pollatos, Azamat Yeldesbay, Arkady Pikovsky, Michael Rosenblum

**Affiliations:** ^1^Health Psychology, Institute of Psychology, University of UlmUlm, Germany; ^2^Department of Physics and Astronomy, University of PotsdamPotsdam, Germany

**Keywords:** time interval reproduction, synchronization, heart cycle, interoception, interoceptive sensitivity

## Abstract

Internal signals like one's heartbeats are centrally processed via specific pathways and both their neural representations as well as their conscious perception (interoception) provide key information for many cognitive processes. Recent empirical findings propose that neural processes in the insular cortex, which are related to bodily signals, might constitute a neurophysiological mechanism for the encoding of duration. Nevertheless, the exact nature of such a proposed relationship remains unclear. We aimed to address this question by searching for the effects of cardiac rhythm on time perception by the use of a duration reproduction paradigm. Time intervals used were of 0.5, 2, 3, 7, 10, 14, 25, and 40 s length. In a framework of synchronization hypothesis, measures of phase locking between the cardiac cycle and start/stop signals of the reproduction task were calculated to quantify this relationship. The main result is that marginally significant synchronization indices (SIs) between the heart cycle and the time reproduction responses for the time intervals of 2, 3, 10, 14, and 25 s length were obtained, while results were not significant for durations of 0.5, 7, and 40 s length. On the single participant level, several subjects exhibited some synchrony between the heart cycle and the time reproduction responses, most pronounced for the time interval of 25 s (8 out of 23 participants for 20% quantile). Better time reproduction accuracy was not related with larger degree of phase locking, but with greater vagal control of the heart. A higher interoceptive sensitivity (IS) was associated with a higher synchronization index (SI) for the 2 s time interval only. We conclude that information obtained from the cardiac cycle is relevant for the encoding and reproduction of time in the time span of 2–25 s. Sympathovagal tone as well as interoceptive processes mediate the accuracy of time estimation.

## Introduction

The perception of time is an important component of human experience; it is essential for everyday activity and for any kind of complex behavior. Despite this fact, the processes underlying the experience of time and the timing of action are only incompletely understood. Wittmann (2009) highlights that—as no sense organ for time perception exists—all sensory modalities are possible entries at the interface of physical time with perceptual time. In this theoretical concept perceptual time is not “isomorphic” to physical time, and many factors, including attention, memory, arousal, cognitive load, ongoing activity, and emotional states, are all potential modulators of time perception (Block et al., 2010). Many different models exist regarding the cognitive and neurobiological mechanisms underlying the experience of time. Some models of time estimation assume existence of an “internal clock” with a pacemaker producing a sequence of time units that are fed into an accumulator (Treisman et al., 1994; Wittmann, 2009). In a variant of those pacemaker–accumulator models, the attentional-gate model (Zakay and Block, 2004), the time units produced are only registered when attention is directed to time.

Recent debate throws a different light on these concepts by assuming that physiological states and emotions associated with changes in physiological states are not only modulators of an assumed neural clock such as attention and working memory, but could function as a timekeeper themselves (Craig, 2009a; Wittmann, 2009). Such a direct link between the perception of time and physiological processes has been proposed by Craig (2009b), who claims that our experience of time relates to emotional and visceral processes because they share a common underlying neural system, the insular cortex and the interoceptive system. Wittmann (2009) follows that, since emotions and physiological states seem so fundamental to the experience of time, it is tempting to assign a pivotal role to these processes related to a core timekeeping system. In line with this conceptualization, it is conceivable that the number and rate of body signals accumulated in the insula over a given time span create our perception of duration. Craig (2009a,b) suggests that the cortical representation of the sentient self in the anterior insular cortex (AIC) is based on the integration of salience across all conditions in the individual's body and in the physical and emotional environment at each moment of time. He further states that the neural substrates responsible for sentience across time are based on the neural representation of the physiological condition of the body, and that the main homeostatic (autonomic) control function for the maintenance of the physiological condition of the body is cardiorespiratory activity (Craig, 2009b).

In this context it is important to emphasize that individuals differ substantially in the ability to perceive ongoing bodily signals (interoception) (Jones, 1994; Wiens, 2005). The extent of an individual's sensitivity to bodily signals can be defined as *interoceptive awareness* or *interoceptive sensitivity (IS)*. IS is often quantified by measuring a person's ability to perceive one's heartbeats accurately (Critchley et al., 2004; Pollatos et al., 2007a). IS is considered to be an essential quantity in many theories of emotions such as that proposed by James or Damasio (James, 1884; Damasio, 1994). The idea that we feel emotions because we perceive our bodily reactions is a core characteristic of these theories suggesting that participants who perceive bodily signals with a high degree of sensitivity should experience emotions more intensely, and vice versa that reduced IS is accompanied with a reduced experience of emotions (James, 1884; Damasio, 1994). This prediction was confirmed in several studies showing an increased subjective affective experience (Pollatos et al., 2005, 2007b) or increased markers of central processing of emotional stimuli in participants with higher scores of IS (Pollatos et al., 2007b).

While Craig's model proposes a close interaction between interoceptive processes and time perception (Craig, 2009a,b; Wittmann and van Wassenhove, 2009) suggesting that our experience of time emerges from emotional and visceral states processed in the insular cortex, there is only sparse empirical evidence underlying this assumption. One recent study by Meissner and Wittmann (2011) demonstrated that individuals' duration reproduction accuracy (using time lengths of 8, 14, and 20 s duration) correlated positively both with cardiac parameters (the slope of cardiac slowing during the encoding intervals) and with individuals' IS supporting the view that autonomic function and interoceptive processes underpin our perception of time intervals in the range of seconds. The idea that rhythms of the body are directly linked to temporal processes in perception was also shown in another study by Iwanaga (1995) reporting that participants' preferred tempo of successive tones was in a harmonic relation (with a ratio of 1:1, 3:2, and 2:1) to individual heart rates as measured during the presentation of the tone sequences. It can be followed that the cardiac rhythm was interrelated (and possibly synchronized) to the tempi of rhythmic tones suggesting that indeed the own cardiac biorhythm is used in a timing task (Iwanaga, 1995).

The fundamental question of *how* internal signals like one's heartbeat could form the building blocks of time perception, can be addressed using the concept of synchronization which may explain the hypothesized relationship between internal signal processing and time perception. Synchronization is a fundamental nonlinear phenomenon, and it plays an important role in various fields of science and engineering (Glass, 2001; Pikovsky et al., 2001; Strogatz, 2003). Moreover, it is found in live systems, being observed on a level of single cells, physiological subsystems, whole organisms, and even on the level of populations (Pikovsky et al., 2001; Strogatz, 2003). Sometimes, this phenomenon is essential for a normal functioning of a system, e.g., for a coordinated motion of several limbs or for the performance of a pacemaker, where many cells fire synchronously, and in this way produce a macroscopic rhythm that governs respiration, heart contraction, etc. Sometimes, the onset of synchrony leads to a severe pathology, e.g., in case of the Parkinson's disease, when locking of many neurons results in tremor activity. Quite often, the functional role of synchrony is yet unknown, e.g., in case of cardiorespiratory coordination (Schäfer et al., 1999; Bracic and Stefanovska, 2000) or in case of mutual entrainment of respiration and locomotion; possibly its appearance is just a manifestation of a general property of self-sustained oscillators to adjust their rhythms due to a weak interaction.

To quantify the level of synchronization of interacting oscillators, one typically uses a *synchronization index* (SI), also known as phase locking value (Astolfi et al., 2009; Lee et al., 2010; Wilmer et al., 2010). Values of the index close to one indicate a strong interdependence between the phases, what is characteristic for synchronous states; in the absence of synchrony the index nearly vanishes. The index operates with the phases of oscillating processes, so a certain pre-processing of original data is needed. Typically, the Hilbert transform or the complex wavelet transform are used for the phase estimation (Pikovsky et al., 2001); there exist also other techniques, suitable for specific signals. Quantification of synchrony, or generally of the interaction strength, by means of synchronization indices (SIs) was suggested in (Tass et al., 1998; Rodriguez et al., 1999; Mormann et al., 2000) with application to e.g., cardiorespiratory coordination (Mrowka et al., 2000) and brain activity (Tass et al., 1998; Rodriguez et al., 1999).

At the moment it is not clear if the effects of cardiac rhythms on the internal clock in time perception exist and are pronounced enough to be measured in a statistically reliable manner. If they exist, they may be reflected in the phase locking of the internal clock with the cardiac rhythm. However, phase locking does not automatically facilitate time reproduction *accuracy*, as even for perfect locking the minimal error will be of the order of one's heartbeat intervals. Since the mechanisms of possible interaction are yet unknown and because of the high interpersonal variability, the “ideal” time interval for interaction between heart rate and time reproduction to occur is a priori unknown. Therefore, we decided to cover a relatively wide span of time interval lengths varying from 0.5 to 40 s. Our hypothesis was that the heart rate does not exactly determine the time estimation but weakly influences it and that this effect may well depend on the interval duration. Therefore, in the current study we quantified this influence with the help of the synchronization concept. Additionally, we hypothesized that interoceptive processes and inter-individual differences in IS affect the time perception accuracy. Whether or not there is also an interrelation with synchronization measures should be analyzed, too.

## Materials and methods

Twenty-three participants [mean age (*M* ± *SD* years) 23.8 ± 3.1; 5 males and 18 females] were recruited from an introductory psychology course and by advertising announcements at the University of Potsdam. All participants were screened for health status using a questionnaire in which common somatic disorders like heart problems, elevated blood pressure, acute or chronic pain, functional aberrations concerning the gastrointestinal system, the kidneys, the bladder, or the thyroidea were included. Participants were excluded if they had a history of any common psychiatric disorder, in particular anxiety disorders or depression (or any other axis 1 disorders) according to the Diagnostic and Statistical Manual of Mental Disorders (American Psychiatric Association, 1994). Drug use (except of contraceptives) was also an exclusion criterion. Experiments were conducted in accordance with the Declaration of Helsinki. Ethical approval from a local ethic board was obtained. All participants gave their written informed consent.

### Procedure outline

Upon arrival at the laboratory room in the Department of Psychology, each participant completed a set of questionnaires. Afterwards, they were fitted with physiological recording equipment for heart rate (Advanced Neuro Technology, ANT, Netherlands). The room was air-conditioned with an average room temperature of 23 degrees Celsius. The experiment started with a 10-min rest period in which the baseline measures were assessed. This period was followed by the IS task. First, IS was assessed using *N* = 4 heartbeat counting trials (varying in length; 25, 35, 45, 60 s) in accordance with the Mental Tracking Method suggested by Schandry (1981). Participants were asked to count their own heartbeats silently and to verbally report the number of counted heartbeats at the end of the counting trial. During heartbeat counting, subjects should not take their pulse or attempt to use other manipulations facilitating the counting of heartbeats. The beginning and the end of the counting intervals were signaled acoustically. IS was estimated as the averaged over *N* trials heartbeat perception score:

$$IS=\frac{1}{N}\sum_{k=1}^{N}\left(1-\frac{\left|N_{k}^{\left(r\right)}-N_{k}^{\left(c\right)}\right|}{N_{k}^{\left(r\right)}}\right)\text{​},$$

where *N*^(*c*)^_*k*_ and *N*^(*r*)^_*k*_ are the numbers of the counted and actually recorded heartbeats within the *k*-th trial.

After the subjects performed the heartbeat perception task, they proceeded with the time interval estimation trial. We assessed heart rate and respiration (using a respiration belt) during the whole experiment. As we searched for a possible phase interdependence between heart rate and time estimation variables, we used a free reproduction task in which participants had to encode the duration of varying time intervals (so-called presentation intervals) in order to reproduce them later (so-called reproduction intervals). In the following we denote the beginning and end points of the presented intervals by PB and PE, respectively. Similarly, the beginning and the end points of the reproduced intervals are denoted by RB and RE. Respiration data during encoding or reproduction was not analyzed in this study.

The length of the time intervals varied between 0.5 and 40 s (0.5, 2, 3, 7, 10, 14, 25, 40 s). We decided to present time intervals with increasing time length (from 0.5 to 40 s) and to decrease time length in a similar fashion (from 40 to 0.5 s). Ten repetitions for each interval length were used. The whole experiment lasted about 40 min.

### Data analyses

First, the time reproduction accuracy was assessed by comparing the estimated time durations with the actual presentation times and calculating an average absolute error score for each of the eight time intervals. We calculated Pearson's correlation coefficient between IS and absolute error scores. Time estimation error scores were analyzed using a repeated-measures analysis of variance (ANCOVA) with the factors *Interval Length* (eight levels) and *IS* as covariate.

**Figure 1:**
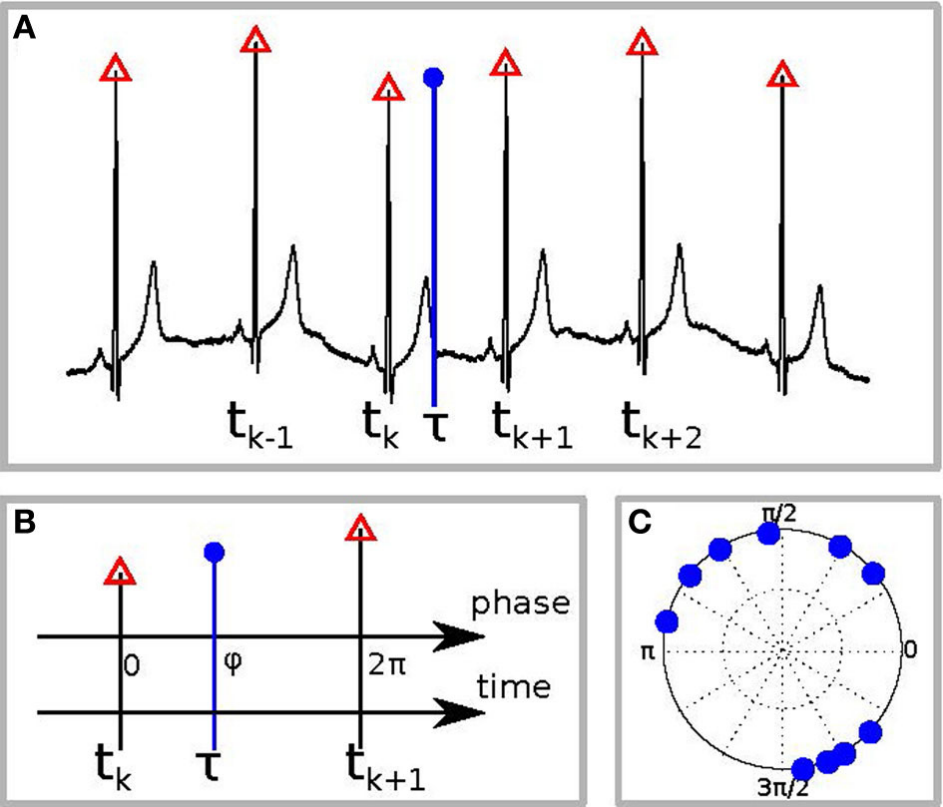
**Schematic illustration of the phase calculation. (A)** The black curve is the ECG, red triangles denote the R-peaks, and the blue vertical circle denotes the time of the response, e.g., of RB or RE. **(B)** Defining the phase of R-peak, preceding the response, as 0 and of the following R-peak as 2π we find the phase of the response φ by linear interpolation. **(C)** An example of the distribution of response phases of RE for 10 different trials.

Second, we tested whether there is interdependence between the phase of the reproduced sequence and the phase of the cardiac cycle. For this goal we first detected all R-peaks in the electrocardiogram and labeled them by corresponding times *t*_*k*_(see Figure 1A). Suppose the event, e.g., pushing the button for the end of interval reproduction, occurred at time τ. To estimate the phase of this event relatively to the cardiac phase, we assigned the phase value φ = 0 to the R-peak preceding the event and the value φ = 2π to the next R-peak. Let these peaks occur at the times *t*_*k*_ and *t*_*k* + 1_. Then the cardiac phase at the instant of the event is obtained via linear interpolation as (see Figure 1B) (Schäfer et al., 1999):

$$\varphi =2\pi \frac{\tau -t_{k}}{t_{k+1}-t_{k}}\text{​}.$$

Repeating the procedure for all *N* trials, we obtain a set of values φ*_j_*, where *j* = 1,…,*N*. It is illustrative to plot them on a circle (an example for one subject is shown in Figure 1C). A deviation of the distribution of φ_*j*_ from the uniform distribution indicates an interrelation between the processes. This interrelation is quantified by the SI:

$$SI=\sqrt{{\left(\sum_{j=1}^{N}\cos(\varphi_{j})\right)}^{2}+{\left(\sum_{j=1}^{N}\sin(\varphi_{j})\right)}^{2}}.$$

All obtained SIs were statistically tested for significance. Therefore, we calculated SIs for every person and for the eight different interval lengths (0.5, 2, 3, 7, 10, 14, 25, 40 s), both for RB and RE events. For each calculation of SI we used 10 trials (see Figure 1C for an example). Totally, with 23 subjects and 8 time intervals, we obtained 184 values. For further statistical analyses we compared the obtained SIs (for each time interval and each person) with the index for randomly distributed points. Since in the real experiment we have (with several exceptions) 10 trials, we have taken 10 points, uniformly distributed between 0 and 2π, and computed the SI. For very large number of randomly distributed points, the index shall tend to zero; since we have only 10 points, this value is typically not small. Repeating this procedure 10,000 times, we obtained the average value 0.28 (recall that by definition the index is positive and, therefore, we obtain a biased estimate) and quantiles of the distribution, which are then used to identify significant cases, see discussion of Table 3 below. Additionally we averaged SIs for every time interval referring to (a) reproduction start and (b) reproduction end. We then calculated a maximum SI for each time interval using the maximum value (referring either to RB or RE) for each individual participant and averaging these scores for the whole sample. We finally checked whether the obtained value is significantly larger than 0.28.

Finally, we computed the most important time-domain measures of the heart rate variability (HRV). For this goal we first obtained all normal interbeat intervals *RR*_*k*_ = *t*_*k* + 1_ − *t*_*k*_ and then computed their average, the standard deviation, and the root mean square of the successive differences (RMSSD). The RMSSD, an indicator of vagal activity, is derived from the HRV as

$$RMSSD=\sqrt{\frac{1}{M-1}\sum_{k=1}^{M-1}{\left(RR_{k+1}-RR_{k}\right)}^{2}},$$

where *M* is the number of *RR* intervals.

All HRV measures were calculated for the baseline period of 5 min and analyzed using repeated-measures analysis of variance (ANOVA) with the factors *interval length* (eight levels). Next, we performed a correlation analysis of IS, SIs, and HRV measures.

## Results

### Time reproduction accuracy, interoceptive sensitivity, and vagal tone

Absolute and relative errors (both time overestimation as well as underestimation) for the eight time interval lengths are depicted in Table 1. All further analyses refer to the absolute error scores.

**Table 1:** **Absolute and relative errors for the different time interval lengths used**.

**Time interval length (in seconds)**	**Absolute error in seconds (±standard deviation, *SD*)**	**Time overestimation**		**Time underestimation**	
		***N***	**Relative error in seconds (±*SD*)**	***N***	**Relative error in seconds (±*SD*)**
0.5	0.09 (±0.06)	13	0.02 (±0.06)	10	−0.05 (±0.03)
2	0.20 (±0.11)	9	0.03 (±0.14)	14	−0.20 (±0.09)
3	0.39 (±0.24)	6	0.32 (±0.28)	17	−0.42 (±0.22)
7	1.09 (±0.76)	3	1.07 (±0.60)	20	−1.10 (±0.75)
10	1.38 (±1.16)	5	0.75 (±0.64)	18	−1.55 (±1.23)
14	2.32 (±1.93)	3	0.49 (±0.68)	20	−1.60 (±1.92)
25	5.51 (±3.79)	2	0.25 (±0.09)	21	−6.01 (±3.58)
40	9.96 (±5.90)	0	−	23	−9.96 (±5.90)

The ANCOVA revealed a significant effect of *Interval Length* [*F*_(*df* = 7.147)_ = 12.64, *p* < 0.001, η^2^ = 0.38, ε = 0.97]. The main effect of IS and the interaction effect were not significant [*F*_(*df* = 1.21)_ = 3.72, *p* = 0.07; *F*_(*df* = 7.147)_ = 2.33, *p* = 0.13]. The mean obtained heartbeat perception score was 0.65 (*SD* 0.19).

The mean heart rate was 66.5 beats per minute (*SD* 9.7 bpm, minimum 49 bpm, maximum 84 bpm). For further analyses we focused on the RMSSD. The RMSSD is sensitive to high-frequency heart period fluctuations in the respiratory frequency range and has been used as an index of vagal cardiac control (Malik et al., 1996; Task Force of the European Society of Cardiology and the North American Society of Pacing and Electrophysiology, 1996). The mean AVNN (average of all NN intervals) was 0.95 s (standard deviation of all NN intervals, SDAVNN = 0.09). The mean RMSSD was 39.2 ms (*SD* 27.2 ms).

**Figure 2:**
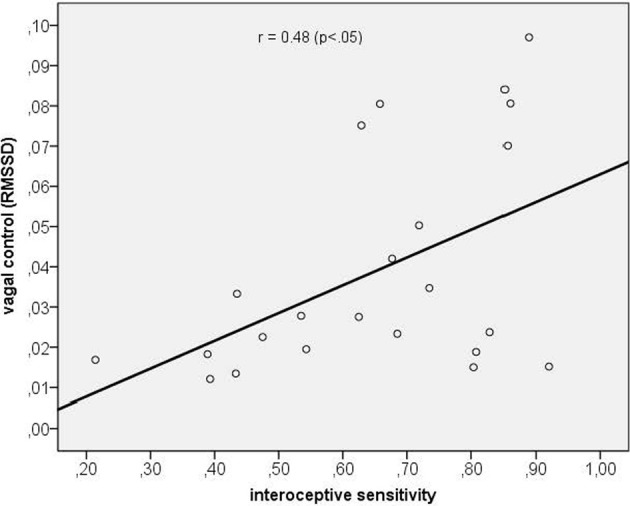
**Scatterplot between interoceptive sensitivity and vagal control as indexed by the RMSSD**.

Next, we assessed the correlation between vagal cardiac control and IS. The correlation coefficient obtained was significantly positive with *r* = 0.48 (*p* < 0.05) indicating that IS was associated with greater vagal control of the heart (see Figure 2).

**Figure 3:**
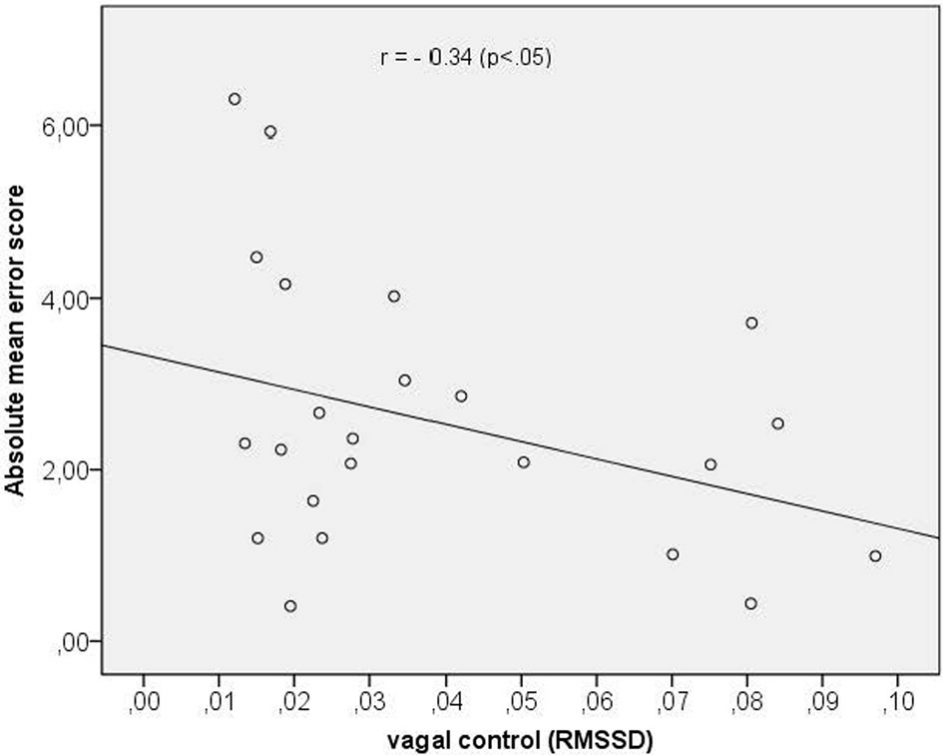
**Scatterplot between vagal control (as indexed by the RMSSD) and absolute mean error score**.

In a last step we assessed the correlation between vagal cardiac control and time perception accuracy (mean score across all time intervals). We obtained a significant negative correlation coefficient of *r* = −0.34 (*p* < 0.05) indicating that a greater vagal control of the heart was associated with a better time reproduction accuracy. The scatterplot is depicted in Figure 3.

### Synchronization analyses of heartbeat cycle and time reproduction variables

Averaged over all subjects SIs for every time interval referring to (a) reproduction start and (b) reproduction end are depicted in Table 2.

**Table 2:** **Statistical analysis of the synchronization indices for the different time intervals**.

**Time interval length (in seconds)**	**Reproduction begin**	**Reproduction end**	**Maximum SI**		
	**Mean SI (*SD*)**	**Mean SI (*SD*)**	**Mean SI (*SD*)**	***T*(***df*** = **23**)**	***p***
0.5	0.30 (0.14)	0.27 (0.11)	0.34 (0.12)	2.59	n.s.
2	0.25 (0.13)	0.28 (0.15)	0.36 (0.14)	1.76	n.s.
3	0.30 (0.15)	0.28 (0.14)	0.38 (0.12)	4.03	^*^
7	0.25 (0.12)	0.26 (0.15)	0.34 (0.12)	2.53	n.s.
10	0.30 (0.17)	0.30 (0.17)	0.38 (0.14)	3.63	^*^
14	0.25 (0.13)	0.30 (0.16)	0.36 (0.14)	2.94	^*^
25	0.27 (0.13)	0.34 (0.15)	0.39 (0.13)	4.23	^**^
40	0.30 (0.15)	0.28 (0.16)	0.36 (0.16)	2.65	n.s.

The second and the third columns show the averaged over 23 participants values for indices, computed for reproduction begin and reproduction end events, respectively. We remind that each index is computed from 10 measurements, obtained from 10 trials. The fourth column shows the mean of the maximal (from two events) index. Notations for the significance level:

* p < 0.05;

** *p < 0.01; n.s. stands for not significant*.

As visual inspection of the data revealed that in several individuals there was a clear pattern toward a high synchronization *either* for RB *or* for RE for one time interval length, we used these indices to assess a maximum SI for each time interval. Corresponding results are also summarized in Table 2.

For further statistical analyses we compared the obtained SI (for each time interval) with the index for randomly distributed points which had an average value of 0.28 (see Methods). We use the distribution of indices for artificially generated surrogate data to test the SIs obtained in experiments for significance, using *t*-tests. Due to multiple comparisons, we used a Bonferroni corrected significance level when applying our analyses to the maximum SI (i.e., *p* < 0.05 corresponds to *p* < 0.05/8 = 0.006; *p* < 0.01 corresponds to *p* < 0.01/8 = 0.001). Using this correction, the SIs were significantly higher than the random distribution score for the time intervals of 3, 10, 14, and 25 s (see Table 2). It should be noted that in this procedure we slightly overestimate the significance because of taking a maximum of SIs for RE and RB.

**Figure 4:**
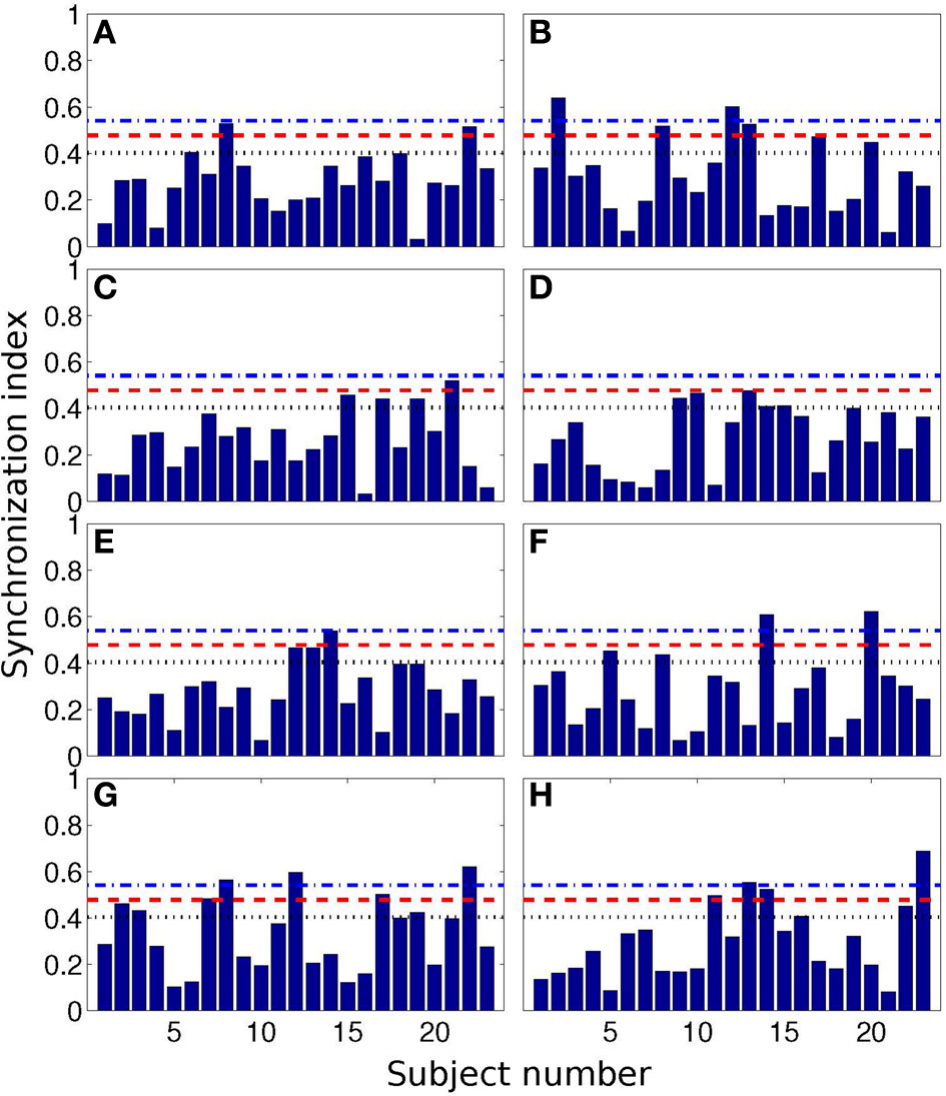
**Synchronization index of the RE response for all 23 participants and for different interval durations: 0.5 s (A), 2 s (B), 3 s (C), 7 s (D), 10 s (E), 14 s (F), 25 s (G), and 40 s (H)**. Horizontal blue dash-dotted line, red dashed line, and black dotted line show 5, 10, and 20% quantile thresholds, correspondingly.

In Figure 4 we show all 184 values of SI. Here we also present the values, corresponding to the 0.05, 0.10, and 0.20 quantiles of the distribution. These threshold values are 0.54, 0.48, and 0.43, respectively; they are depicted as horizontal lines.

These results are also illustrated in Table 3, where we show the number of the cases when SIs were larger than the threshold values.

**Table 3:** **The number *N_q_* of cases out of 23 (number of subjects) when the synchronization indices for the RE and RB events were larger than the corresponding 0.05, 0.1, and 0.2 quantile threshold values (see also Figure 4), obtained for randomly distributed points on a circle**.

**Time interval length (in seconds)**	**Reproduction begin**			**Reproduction end**		
	***N*_0.05_**	***N*_0.1_**	***N*_0.2_**	***N*_0.05_**	***N*_0.1_**	***N*_0.2_**
0.5	1	1	2	0	2	3
2	0	1	3	2	4	6
3	2	3	3	0	1	4
7	1	1	2	0	0	5
10	4	5	5	1	1	3
14	1	1	2	2	2	4
25	1	1	4	3	5	8
40	1	4	6	2	4	6

From the Table 3, one can see that for time interval 25 s and for RE event, 3, 5, and 8 subjects out of 23 reached significance level of 5, 10, and 20% quantile, correspondingly. RE event for time intervals 2 and 40 s is also characterized by high SIs (6 out of 23 for 20% quantile).

**Figure 5:**
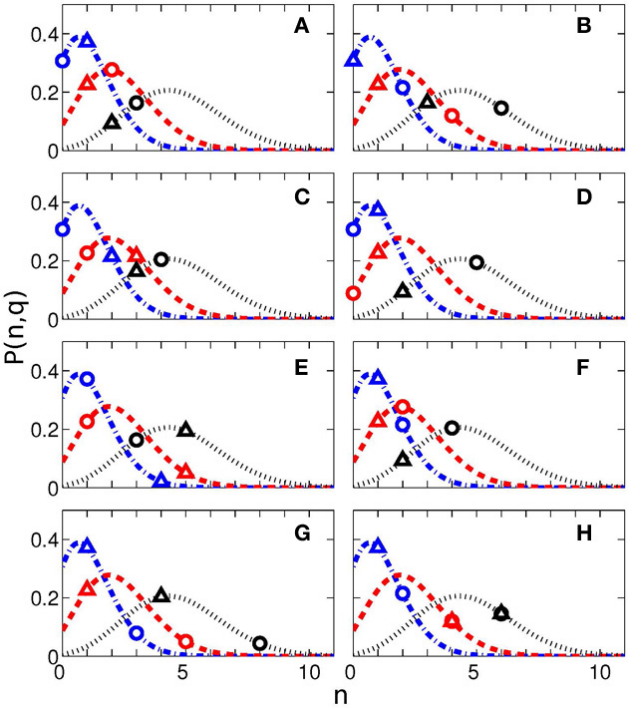
**Probability to have *n* cases with probability *q* within *N* measurements (see Equation 1) for different interval durations: 0.5 s (A), 2 s (B), 3 s (C), 7 s (D), 10 s (E), 14 s (F), 25 s (G), and 40 s (H)**. The blue dash-doted, red dashed, and black dotted lines correspond to the probability *q* = 0.05, *q* = 0.1, and *q* = 0.2. The experimentally obtained values of *N_q_* (see Table 3) and corresponding probabilities are shown by triangles (for reproduction begin) and circles (for reproduction end). The total number of measurements for each time interval is *N* = 23.

Further analysis of the significance of the synchronization analysis is performed by means of comparison of our results with an amount of cases obtained in a random distribution model. We used the following formula of the probability to have *n* events with the probability *q* within *N* measurements

$$P(n,q)=C_{N}^{n}{\left(1-q\right)}^{\left(N-n\right)}q^{n},$$

where *C^n^*_*N*_ is the binomial coefficient. The probabilities *P*(*n*,*q*) for *q* = 0.05, *q* = 0.10, and *q* = 0.20 for *N* = 23 measurements for each of eight time intervals are shown in Figure 5 by lines. The values of *N_q_* actually obtained for these quantiles are shown by symbols. If these symbols are situated close to the maxima of the probability curves, then the results are indistinguishable from the random distribution and thus non-significant. On the contrary, if the symbols are positioned on the tail of the distribution, then the indices are larger than can be expected for the random distribution. The difference from the random case is especially pronounced for the RE events for 25 s interval.

As follows from Figure 5G, the difference from the random case is especially pronounced for the RE events for 25 s interval, what corresponds to the result in Table 3, with 8 subjects out of 23 reaching the significance with 20% quantile.

**Figure 6:**
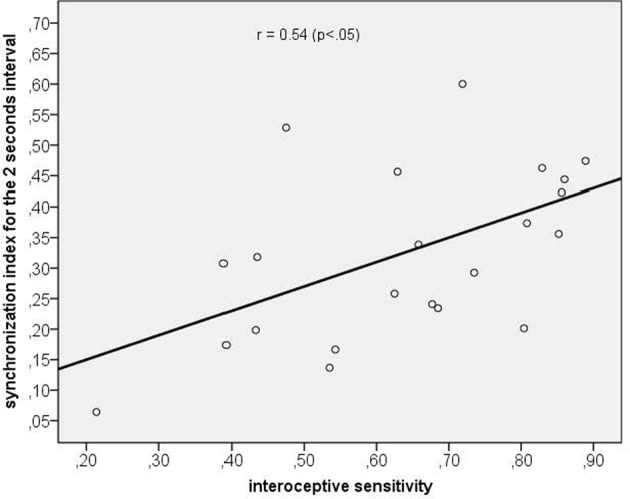
**Scatterplot between interoceptive sensitivity and the maximum synchronization index for the 2 s time interval**.

Using correlation analyses we tested whether a higher synchronization was associated with (a) corresponding time reproduction accuracy and (b) IS. Time reproduction accuracy was not significantly correlated with any corresponding SI (maximum SI used), while the only significant correlation we obtained was between IS and the maximum SI of the 2 s time interval (*r* = 0.54, *p* < 0.05, *p*-value Bonferroni corrected; see Figure 6).

## Discussion

The present study shows that the cardiac rhythm affects time perception. We observed that information obtained from the cardiac cycle influences the encoding and reproduction of time as demonstrated using synchronization analyses. As hypothesized, the average SIs between start/stop points of duration reproduction and heart rhythm were marginally significant compared to a random distribution in the whole sample for time intervals of 3, 10, 14, and 25 s length. On average, we did not observe a significant synchronization between heart rate and time reproduction responses for intervals of shorter as well as of longer duration. However, for individual persons we observed significant synchrony for intervals of 2 and 25 s length. In accordance to prior studies using the temporal reproduction method in the multiple-seconds range (e.g., Meissner and Wittmann, 2011) time lengths reproduced were in the mean shorter than physical time (except of the 0.5 s interval length).

Taking the mean heart rate during baseline (mean 66 beats per minute) as reference it can be followed that intervals covering the amount of at least three heart cycles up to a maximum of 30 heart cycles are mostly favorable for the occurrence of interaction and therefore for observation of synchronization between heart beats and time reproduction. Nevertheless, individual heart rates varied between participants substantially (from 49 to 84 bpm). Therefore it is difficult to exactly interfere the ideal time span during which information of the heart cycle could be used for time estimation. Having in mind these points it can be concluded that time lengths between 3 and 25 s are presumably lying within these optimal preconditions and these were also the time lengths with significant SIs as assessed in in our sample. Future studies could solve this problem by using online assessed individual heart rates and adjusted interval lengths that cover whole multiples of individual heart cycle lengths.

Referring back to the high variance between participants in baseline heart rates it is conclusive that on an individual level significant synchronization can be found only for certain time lengths and for a varying percentage of individuals. Having in mind that we assume an interaction between the heart cycle and time estimation, an interval length that is close to a whole multiple of the individual heart cycle length (e.g., 3 s correspond to three heart cycles if the heart rate is 60 bpm and to four cycles if the heart rate is 80 bpm) is a more suitable precondition to observe a statistically evident synchronization in this subject. Preliminary data analysis of the 3 s interval could partly support this idea by showing that two out of three participants with heart rates of exactly 60 bpm respectively 80 bpm descriptively exhibited a higher SI (maximum synchronization score; single scores 0.52, 0.39, and 0.33) as compared to the mean SI of the whole sample (mean score 0.38). And there are yet two other sources of inter-individual variance to be taken into account, namely vagal control of the heart and IS.

We obtained a significant positive correlation coefficient between IS and the maximum SI of the 2 s time interval, indicating that participants with higher IS show a higher degree of phase locking between heart cycle information and time reproduction start/stop responses. While Meissner and Wittmann (Meissner and Wittmann, 2011) could demonstrate that IS was associated with time reproduction accuracy in the multi-second range, we now observe a significant modulation of synchronization processes for the shorter time range of 2 s. It is an important fact to note that this interval length was also one length in which we observed significantly many individual cases with high SIs. Interoceptive processes and individual sensitivity to interoceptive signals like the heart beat are variables that might explain part of the observed variance in synchronization measures.

Additionally, we found that sympathovagal tone as operationalized by the RMSSD mediates the *accuracy* of time estimation. A greater vagal control of the heart, i.e., greater RMSSD, was associated with better mean time reproduction accuracy. This observation is in accordance to the model of neurovisceral integration proposed by Thayer and Brosschot (2005). Within this model it is hypothesized that a higher sympathetic activation is linked to hypervigilance and inefficient allocation of attentional and cognitive resources (Thayer and Brosschot, 2005), while a greater vagal tone was shown to be associated with efficient attentional regulation, response flexibility (Friedman and Thayer, 1998; Elliot et al., 2011) and efficient emotion regulation (Elliot et al., 2011). Our data support the idea that a higher vagal tone might also facilitate the allocation of attention resources involved in time estimation.

Confirming this assumption, Meissner and Wittmann (2011) could demonstrate that individuals' duration reproduction accuracy correlated positively with the vagal-driven slope of cardiac slowing during the encoding of time interval. It is important to notice that our data also showed that IS was associated with a greater vagal control of the heart. It can be followed that—similar to other cognitive tasks—a higher vagal tone advantages the detection of internal signals such as the heart beats. Referring back to our results, we assume that there are critical time lengths in which both processes—vagal control of the heart and interoceptive processes—might also lead to contradictive effects on time reproduction accuracy.

Coming back to the question of an interrelation between synchronization and time estimation accuracy, we did not observe significant correlation between time reproduction accuracy and the degree of synchronization, as has been hypothesized. We therefore found evidence for our hypothesis that the heart rate influences, but does not exactly determine time estimation. Indeed, assuming for example a high degree of synchronization, i.e., a pronounced phase locking, this high synchronization would only then facilitate time reproduction accuracy if the time interval to be reproduced is a whole multiple of the individual cycle length. If, on the contrary, the actual length of the interval is 2.5 times of the individual cycle length and this subject reproduces a length corresponding to two or three times of the individual cycle duration, the reproduction error will be quite high. We therefore assume that synchronization processes reflect a mechanism that might be a systematic source of “errors” in timing tasks as e.g., demonstrated by Iwanaga (1995). The latter study could show that the tempo of represented successive tones was systematically changed into a harmonic relation (1:1, 3:2, 2:1) to the participants' individual heart rates supporting our idea that one's own cardiac biorhythm is used in a timing task as demonstrated in the current study.

Our results highlight that the cardiac cycle and information obtained from cardiac rhythm might underpin our perception of time intervals in the range of seconds as proposed in several theoretical approaches of time perception (see e.g., Craig, 2009b; Wittmann, 2013). One important model to explain the internal representation and reproduction of temporal durations in the supra-second range and was introduced by Wackermann and Ehm (2006). Referring to our study, bodily processes like the heart beat can be interpreted as one possible inflow unit. The dual klepsydra model (DKM; klepsydra: Greek for water clock) assumes that subjective duration is represented by the states of inflow–outflow units, which function as leaky integrators (as described by Wittmann, 2013). These units can be thought to function like water clocks, with water flowing in at a constant rate and simultaneously flowing out (the “leakage”) at a rate proportional to the momentary accumulated state. Wittmann emphasizes that the state of the integrator is thus a nonlinear (climbing) function of physical time (Wittmann et al., 2010).

The DKM has been discussed as being an intrinsic model for the integration of bodily signals for the representation of time in the supra-second range (Sysoeva et al., 2011; Wittmann et al., 2011; Wittmann, 2013). In line with this idea Wittmann et al. (2010) presented fMRI evidence that an accumulation function in the posterior insula exists and might be correlated with the encoding of time intervals using a temporal reproduction task. Importantly, the authors assumed that—given the close connection between the insular cortex and ascending body signals—the accumulation of physiological changes in body states is the basis for subjective duration (Wittmann et al., 2010; Wittmann, 2013). Wittmann suggests that intrinsic processes for the representation of the bodily self—like a better access to visceral feedback and ascending signals from the heart as measured by IS—might additionally serve as a means to represent time (Wittmann, 2013).

Some shortcomings have to be noticed. First, we assessed a rather small sample size of young and healthy participants. It is necessary to re-assess synchronization measures in a larger sample and also to systematically include interindividual variance concerning IS in the composition of such a sample. Second, we tried to cover a large time span and therefore sacrificed the amount of repetitions used for each time length we used. Future studies could benefit from more elaborative focus on interval lengths between 2 respectively 3 and 25 s as we found most pronounced results within this range. Using more repetitions and more participants as well as using experimental designs with online assessed individual heart rates and adjusted interval lengths will help to clarify our preliminary results and to disentangle the complex result pattern found in order to verify both the technique of synchronization analysis in time perception as well as its interaction with bodily signals. And third, other slower biorhythms like respiration have to be included and experimentally manipulated next to heart rate to get a more definite picture of the interaction between bodily rhythms and our experience of time.

We conclude that the heart and information from the heart cycle could serve as input signals used for the reproduction of time intervals in the range of several seconds. Our results highlight one important mechanism of the embodiment of time. Further research with different time perception tasks and more participants are needed to follow this important research and avenue new aspects of fundamental principles in time perception.

### Conflict of interest statement

The authors declare that the research was conducted in the absence of any commercial or financial relationships that could be construed as a potential conflict of interest.
